# Karimabad virus: A neglected sandfly-borne pathogen

**DOI:** 10.1097/MD.0000000000048467

**Published:** 2026-05-08

**Authors:** Yingxin Tu, Kai Guo, Meixi Ren, Anan Wang, Tao Xu, Jian Song, Guoyu Niu

**Affiliations:** a School of Public Health and School of Life Sciences and Technology, Shandong Second Medical University, Weifang, China; b Department of Business Management, Yantai Center for Disease Control and Prevention, Yantai, China.

**Keywords:** Karimabad virus, phlebovirus, sandfly, sandfly-borne viruses

## Abstract

Karimabad virus (KARV), a member of the *Phlebovirus* genus within the *Phenuiviridae* family, is primarily transmitted through the bites of sandflies *(Diptera: Psychodidae*). KARV has a wide geographic distribution, spanning the Mediterranean region, Africa, Central Asia, and West Asia, with recent studies identifying its presence in the Xinjiang region of China. Despite its potential public health significance, there is currently a paucity of research focusing on KARV. This review provides a comprehensive analysis of KARV’s molecular biological characteristics, epidemiological features, cross-host transmission potential, and research methods to enhance understanding and guide future research endeavors. Serological studies have detected KARV-specific antibodies in wild and domestic animals, as well as in human populations residing in endemic areas. Phylogenetic analyses suggest that KARV exhibits genetic diversity, which may be associated with the biological characteristics and immune responses of its hosts. KARV represents a virus with potential public health significance that requires enhanced research focus. Current knowledge gaps exist regarding this understudied phlebovirus, highlighting the need for future research endeavors to better understand its characteristics and implications.

## 1. Introduction

The *Phlebovirus* genus encompasses a diverse group of viruses primarily transmitted by sandflies, mosquitoes, ticks, and other arthropod vectors.^[1,2]^ According to the most recent classification guidelines provided by the International Committee on Taxonomy of Viruses, the *Phlebovirus* genus comprises 67 recognized species, exhibiting a wide geographic distribution spanning Southern, Southeastern and Central Europe, Africa, Central Asia, and West Asia.^[3–5]^ Phleboviruses can cause a spectrum of clinical manifestations, ranging from mild self-limiting febrile illnesses to severe neuroinvasive infections, collectively referred to as sandfly fever.^[6]^ Numerous viruses within the *Phlebovirus* genus, such as Toscana virus, Sicilian virus, Naples virus, and Karimabad virus, are capable of causing sandfly fever. Although serological evidence suggests human infection with Karimabad virus (KARV), no clear clinical manifestations associated with KARV have been reported to date. In 1959, researchers first isolated and identified KARV from sandflies collected in the Isfahan region of Iran.^[7]^ However, subsequent genomic and antigenic analyses revealed that despite sharing some biological similarities, KARV is genetically distinct from Gabek Forest virus. Consequently, these viruses were initially classified as a novel species complex, designated as the Karimabad species complex.^[8]^ With advancements in virology and molecular biology, the International Committee on Taxonomy of Viruses no longer supports the classification of species complexes based solely on serological relationships. Currently, viral classification relies more heavily on molecular phylogenetic analysis, which provides a more accurate scientific basis for taxonomy. Therefore, based on current taxonomic criteria, KARV and Gabek Forest virus should be considered distinct virus species rather than part of a species complex.

The ability to transmit across various host species is a crucial indicator of a virus’s transmissibility, which is closely linked to its pathogenicity and potential to cause widespread outbreaks. The diverse transmission routes and wide range of host species associated with KARV underscore its complex ecological role in nature. KARV not only establishes a transmission cycle between specific sandfly vectors and great gerbil reservoirs but also exhibits the potential for cross-species transmission, rendering humans and other wild animals as potential hosts. The cross-host transmission capability of KARV, combined with the uncertainty surrounding the virus’s incubation period, presents significant challenges for early diagnosis and prevention efforts. Moreover, as global warming trends continue, the geographic range of sandfly vectors may gradually expand, and their population density may increase, potentially amplifying the spread of KARV. By providing a comprehensive analysis of the molecular biological characteristics, epidemiological features, and dynamic changes of KARV across diverse ecosystems, this review synthesizes current knowledge on how these factors influence the virus’s transmission patterns and host range. The review aims to establish a novel theoretical framework for understanding KARV while offering evidence-based guidance for the development of effective public health interventions.

## 2. Virological characteristics

### 2.1. Virus structure

**Figure 1.**
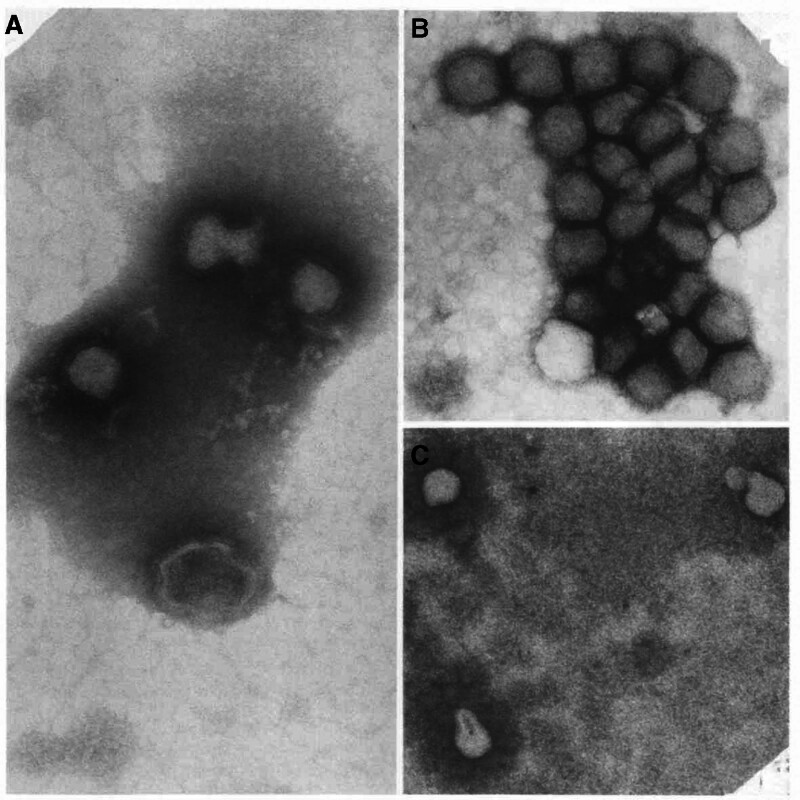
Electron micrograph of Karimabad virus negatively stained with 2% sodium phosphotungstate, showing spherical virions approximately 100 nm in diameter with prominent surface projections. Scale bar = 100 nm. Reprinted from Robeson G, et al, J Virol. 1979;30:339–350, Figure 8A-B, with permission from the American Society for Microbiology (License #1646565-1).

**Figure 2.**
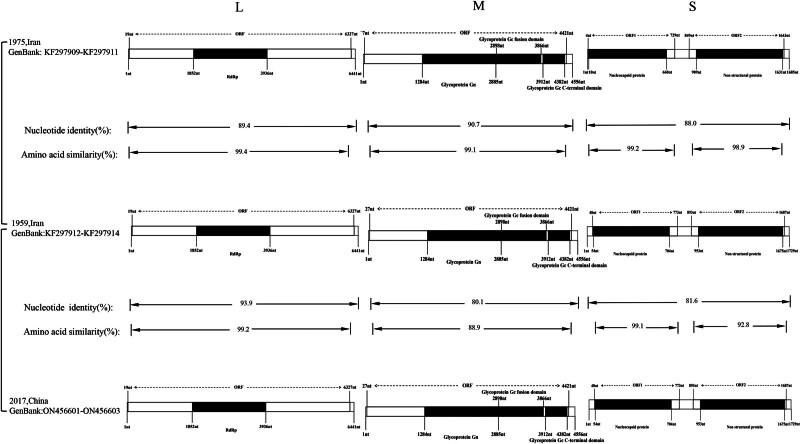
Schematic representation of the genome organization of KARV. KARV = Karimabad virus.

Despite the successful isolation of KARV in 1959 and 1975, the passage of time has left us without original electron-microscopy images; the only available ultrastructural data are those reported by Robeson et al (1979), who described roughly spherical virions of 100 ± 10 nm in diameter bearing 10 nm surface projections^[9]^ (Fig. 1). Within the *Phenuiviridae* family, virus particles typically exhibit a spherical morphology, ranging from 80 to 120 nm. The surface of the virus particles is generally covered with heterodimers composed of Gn and Gc glycoproteins, which form spikes protruding from the viral envelope. These surface spikes play crucial roles in virus recognition, host-cell entry and the assembly and release of virus particles.^[8,10]^ As a member of the *Phlebovirus* genus, KARV possesses a genome consisting of 3 single-stranded negative-sense RNA segments: large (L), medium (M), and small (S), which encode various structural and nonstructural proteins.^[11,12]^ The L segment, 6441 bp in length, encodes the RNA-dependent RNA polymerase (RdRp); the M segment, 4556 bp, encodes the glycoproteins Gn and Gc; and the S segment, 1685 bp encodes the nucleocapsid protein (N) and the nonstructural protein (NSs).^[13,14]^ Research has revealed that the S segment of KARV harbors 2 open reading frames: the first ORF, situated at the 3’ end and 726 nt long, encodes the N protein consisting of 241 amino acids; the second ORF, spanning 789 nt via an ambisense strategy, encodes the NSs protein composed of 262 amino acids^[14]^ (Fig. 2). These structural features provide a molecular basis for the classification of KARV within the *Phlebovirus* genus and are likely to be associated with its specific functions in the viral life cycle.

### 2.2. Virus replication process

The replication cycle of KARV is likely to possess certain unique features that distinguish it from other viruses within the *Phenuiviridae* family. While the initial infection steps of KARV resemble those of other *Phenuiviridae* viruses, involving binding to host cell receptors and facilitating the fusion of virus particles with the cell membrane, the specific cellular receptors utilized by KARV remain unidentified. However, studies of closely related phleboviruses provide insights into potential mechanisms. Recent research has demonstrated that Rift Valley fever virus (RVFV) Gn glycoprotein directly binds to low-density lipoprotein receptor-related protein 1 (LRP1), serving as a critical host entry factor.^[15]^ Similarly, other bunyaviruses including Oropouche orthobunyavirus utilize LRP1 for cellular entry,^[16]^ suggesting this may represent a conserved mechanism within the Bunyavirales order. We hypothesize that the broad host range of KARV may be facilitated by several factors, potentially including: molecular adaptability of the M segment-encoded glycoproteins, which likely target conserved mammalian receptors such as LRP1; the utilization of generalist sandfly vectors (Phlebotomus spp.) capable of vertical transmission; and cellular permissiveness enabled by conserved viral entry pathways shared among phylogenetically distant hosts. However, direct experimental evidence is required to confirm these mechanisms. Although direct experimental validation for KARV is pending, the structural conservation of the Gn glycoprotein suggests a receptor-binding mechanism similar to that of RVFV, facilitating cross-species spillover.^[17]^ Whether KARV employs similar LRP1-mediated entry or utilizes alternative receptor mechanisms requires experimental investigation. Following receptor engagement, glycoproteins undergo pH-dependent conformational changes within endosomal compartments, triggering membrane fusion mediated by the Gc protein.^[18]^ The RNA-dependent RNA polymerase (RdRp) of KARV may exhibit unique transcriptional regulatory mechanisms that enable efficient replication across diverse cellular environments, potentially involving species-specific host factor recruitment or evasion of species-restricted innate immune responses. Post-translational modifications of Gn and Gc glycoproteins, including N-linked glycosylation patterns, are processed by host cellular machinery and may vary between species, influencing viral particle stability, immunogenicity, and transmission efficiency. The assembly and budding processes involve precise coordination between viral structural proteins and host cellular pathways, with species-specific variations in cellular trafficking mechanisms potentially influencing viral particle maturation and release kinetics.

### 2.3. Pathogenesis and clinical presentation

Karimabad virus (KARV) demonstrates an exceptional dissociation between serological evidence of infection and clinical disease severity. Epidemiological surveillance across endemic regions reveals neutralizing antibodies in 66.4% (266/402) of human residents in Isfahan Province, Iran, with no documented cases of severe illness among seropositive individuals.^[19]^ This consistent pattern of subclinical infection extends to animal reservoirs, including 31.6% (12/38) of wild Rhombomys opimus gerbils in Iran,^[19]^ 11.8% (6/51) of Tatera indica rodents in Pakistan,^[20]^ and 2.3% (8/349) of R. opimus in China,^[21]^ all exhibiting seroconversion without observable morbidity.

The molecular basis for this attenuated phenotype – characterized by high seroprevalence without severe morbidity – likely involves specific genomic features. Specifically, naturally occurring truncations in the NSs protein (101-270 aa deletions) may significantly impair the virus’s ability to suppress the host interferon response,^[12]^ leading to rapid viral clearance before severe symptoms develop. Furthermore, the structural protein divergence (79.1–93.9% identity) compared to pathogenic phleboviruses may result in altered tissue tropism,^[8]^ restricting infection to the spleen rather than vital organs like the liver.^[21]^

Three fundamental questions remain unresolved: first, the complete absence of mortality records despite decades of multi-species surveillance; second, the striking disparity between high seroprevalence (up to 66.4%) and lack of severe case reports; third, the undefined parameters of infection kinetics including incubation period and viral load progression. Addressing these gaps requires establishing standardized case definitions for human surveillance and developing controlled animal models using natural host species under biosafety level-3 containment, which would enable definitive characterization of KARV’s pathogenic potential.

## 3. Epidemiology

### 3.1. Vector and host

**Figure 3.**
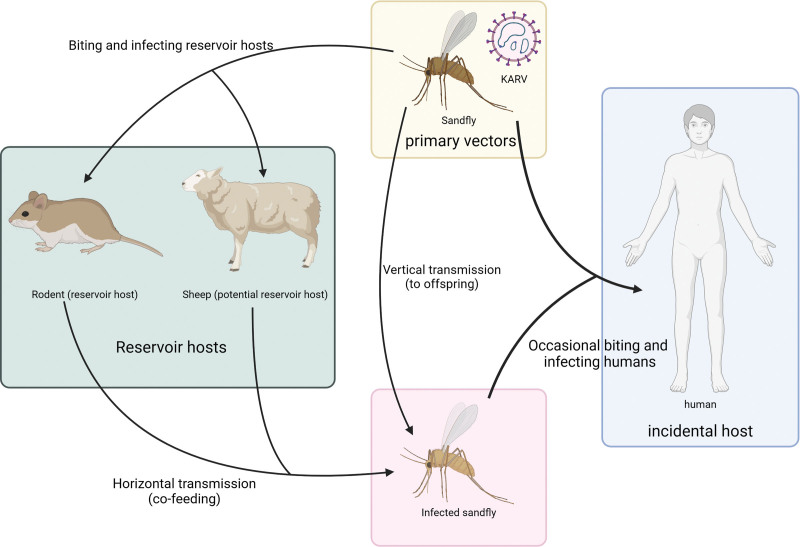
This diagram illustrates the transmission cycle of the KARV virus. The main vector of the KARV virus is the sandfly, which is transmitted to different hosts through bites. Storage hosts include rodents (e.g., mice) and potential storage hosts (e.g., sheep). The virus infects reservoir hosts through the bite of a sandfly and can be transmitted horizontally (by shared feeding) between reservoir hosts. Infected sandflies can also transmit the virus to their offspring through vertical transmission. Occasionally, sandflies bite and infect humans (accidental hosts), resulting in the spread of the virus among humans. The arrows in the diagram illustrate the path of virus transmission between different hosts and vectors. Ke L. Medicine. Created in BioRender; 2026. https://BioRender.com/i25s969. KARV = Karimabad virus.

Sandflies serve as the principal vectors for KARV transmission. Research has established that *Phlebotomus papatasi* and *Phlebotomus perfiliewi* are competent vectors for KARV.^[7]^ While the sandfly species harboring KARV differ across geographical regions, *Phlebotomus papatasi* is regarded as the most significant vector owing to its extensive distribution and elevated infection rate. KARV exhibits the ability to infect a diverse range of mammalian hosts. Great gerbils (*Rhombomys opimus*) in Iran and China,^[19,21]^ *Rattus rattus* and *Rattus norvegicus* in Pakistan, as well as *Tatera indica*, *Meriones hurrianae*, and sheep in the Nile Delta region of Egypt have all been found to be seropositive for KARV.^[20]^ These findings suggest that KARV possesses a broad host range and may pose a potential threat to the health of these animal populations. In nature settings, KARV sustains a complex transmission cycle, primarily involving sandflies and rodents, with occasional spillover events leading to human infections (Fig. 3).

### 3.2. Transmission routes

The primary transmission route of KARV is through the bites of specific sandfly species, such as *Phlebotomus papatasi*. This transmission mode is closely associated with the biological characteristics and geographical distribution of these sandfly vectors. Furthermore, studies have revealed that KARV transmission among sandfly populations may involve 2 distinct modes: vertical transmission and horizontal transmission. Vertical transmission refers to the ability of female sandflies to directly transmit the virus to their offspring, representing a transgenerational transmission mode.^[22]^ Horizontal transmission occurs between sandflies when they co-feed on the blood of a host infected with or immune to KARV, enabling the virus to directly spread among the sandfly population. Through this “sandfly-host-sandfly” cycle, KARV enhances its chances of dissemination within the sandfly population (Fig. 3).

### 3.3. Geographical distribution

**Figure 4.**
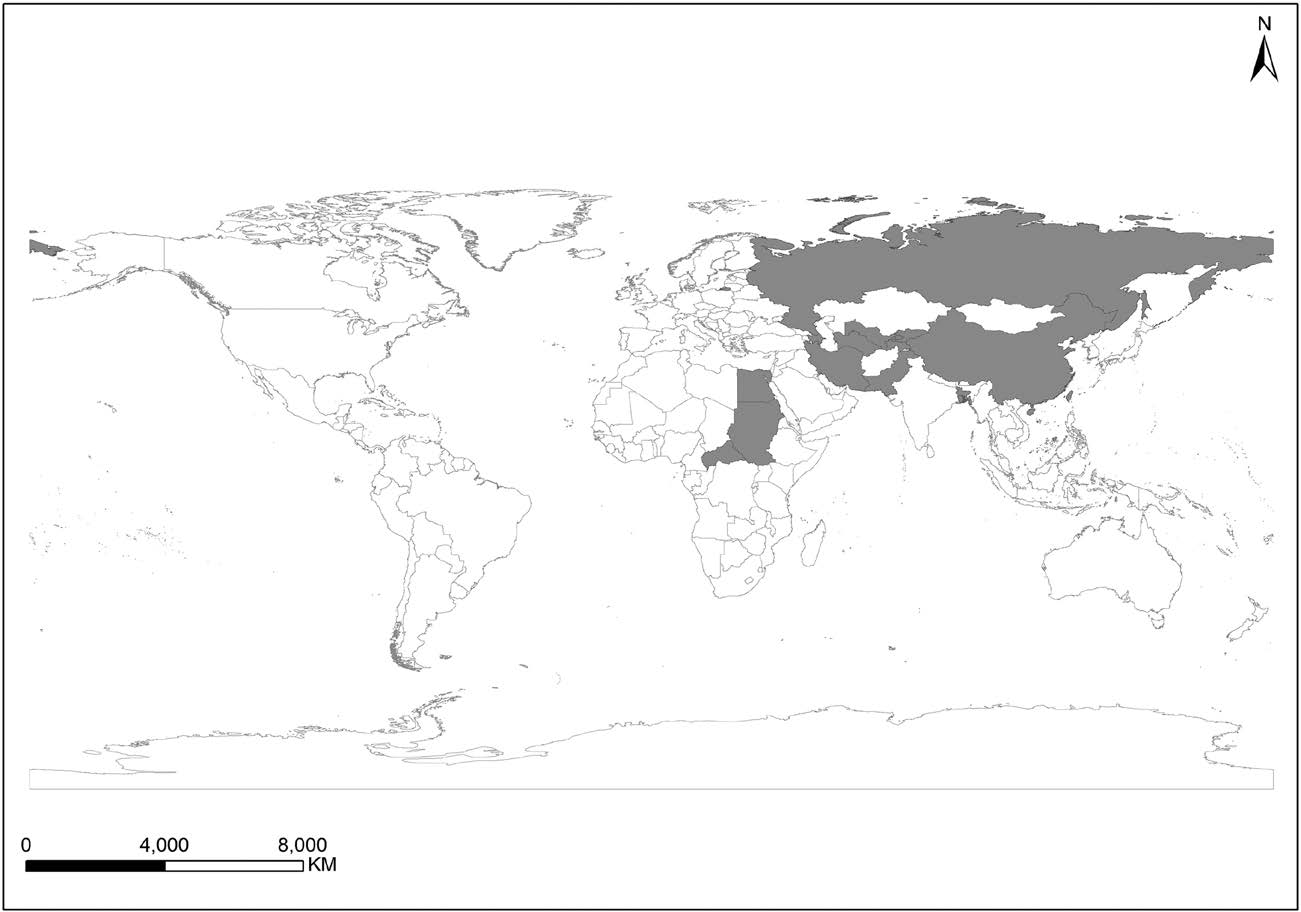
Countries reporting KARV human infections or found in animal hosts as of 2024 (The map was created by the authors using ArcGIS 10.8 software, with base layers sourced from the National Geographic Data Cloud public data repository https://www.resdc.cn/data.aspx?DATAID=205. No copyright permission was required for this use). KARV = Karimabad virus.

KARV was initially discovered in the Mediterranean region, with its first isolation and identification occurring in Isfahan Province, Iran, in 1959. Following its initial discovery, the virus disseminated to multiple regions within Iran, including Isfahan in the central region, as well as Tehran, Khorasan Province, and Razavi Khorasan Province in the northern part of the country.^[6,13]^ With the advancement of research, it became evident that the distribution range of KARV had expanded to countries and regions surrounding Iran. Between 1959 and 2013, the presence of KARV was successively reported in several countries, including Azerbaijan, Uzbekistan, Turkmenistan, Kyrgyzstan, Tajikistan, and Russia.^[8]^ In 2017, Chinese researchers conducted a viral screening of wild small mammals across 7 ecological zones in China, leading to the first confirmation of KARV presence in the country, specifically in great gerbil samples captured in Xinjiang.^[21]^ Apart from the aforementioned regions, KARV has also been reported in the Aswan region of southern Egypt and Sudan.^[8,23]^ Advances in arbovirus research, coupled with the widespread application of next-generation sequencing technology, are expected to further elucidate the geographical distribution of KARV, enabling a more accurate assessment of its epidemic risk and public health implications (Fig. 4).

## 4. Virus isolation

Virus isolation is a crucial foundation for research on KARV. Since the initial isolation and identification of KARV from sandfly samples in Iran in 1959, researchers have continuously optimized virus isolation techniques in subsequent studies to enhance isolation efficiency and sensitivity.^[7,13]^ In 1975, Tesh et al successfully re-isolated KARV in Isfahan Province, Iran, confirming the virus’s existence.^[19]^ The researchers collected a large number of sandfly samples using night trapping and early morning direct collection methods. They then performed virus isolation in the laboratory using the Ten Broeck tissue homogenization technique and Vero cell culture method. This study provided crucial experimental data and methodological references for further research on KARV, while also accumulating experience for subsequent virological research and disease prevention and control. The researchers also discovered that the test tube culture method had higher sensitivity for primary virus isolation. In contrast, the microplate culture system had difficulty distinguishing between cell monolayer defects and viral plaques, making it unsuitable for sandfly samples with low virus titers. This finding is highly significant for optimizing virus isolation methods. Furthermore, the research team successfully isolated 11 strains of KARV from 12,485 sandfly samples, with one strain obtained from a male sandfly sample.^[13]^ This discovery suggests that KARV might be maintained in sandfly populations through vertical transmission,where the virus is transmitted to the next generation by infected female flies via eggs. However, the possibility of horizontal transmission cannot be excluded, whereby the virus spreads between sandflies of the same generation through mating or co-feeding behavior. In summary, the continuous optimization and improvement of virus isolation techniques have laid the foundation for in-depth research on the biological characteristics, transmission mechanisms, and host interactions of KARV.

## 5. Molecular biology research

### 5.1. Nucleic acid detection techniques

Between 2019 and 2020, researchers gathered 29,216 sandfly samples from 7 sampling locations in Iran and utilized molecular identification techniques for analysis. Reverse transcription polymerase chain reaction (RT-PCR) identified 4 super pools as positive for sandfly-borne virus. Subsequent analysis demonstrated that only 2 *Phlebotomus papatasi* sandflies and one *Phlebotomus perfiliewi* sandfly collected from Tehran tested positive for KARV, all of which were non-blood-fed.^[14]^ These findings imply that KARV can be detected in various sandfly species (*Ph. papatasi* and *Ph. perfiliewi*), suggesting that the virus may have a wide sandfly host range. Furthermore, the geographical distribution of positive samples illustrates the presence of KARV in the Tehran region, with positive sandflies identified in 2 consecutive years, 2019 and 2020, suggesting a potential establishment of the virus within this area, contributing to our comprehension of the virus’s geographical distribution. Remarkably, all KARV-positive samples were obtained from non-blood-fed sandflies, implying that they may be more vulnerable to KARV infection. This vulnerability may be ascribed to several factors: Blood-fed sandflies may digest and eliminate the virus after blood-feeding, while non-blood-fed sandflies may not efficiently clear the virus, permitting it to persist and replicate within their bodies; Non-blood-fed sandflies may become vectors of virus transmission through mating with male sandflies, which may transpire during the mating and oviposition period or through other forms of contact transmission; and Non-blood-fed sandflies may play a pivotal role in the KARV transmission cycle as potential vectors from infected hosts to new hosts, contributing to the virus’s ecology and transmission patterns. From September 2013 to June 2021, as part of the China Wild Small Mammal Pathogen Identification Project, researchers captured 1713 wild small mammals in 7 provinces and autonomous regions (Heilongjiang, Xinjiang, Inner Mongolia, Henan, Jiangsu, Guangdong, and Yunnan) and collected spleen samples using the clip method, a nondestructive sampling technique employed to collect spleen samples from live animals. It entails utilizing a small clip or biopsy tool to remove a small piece of tissue without causing substantial harm to the animal, enabling the detection of pathogens such as KARV in a minimally invasive manner.^[21]^ Morphological identification, PCR, and sequencing methods verified that only 8 (2.29%) great gerbil spleen samples captured in the Xinjiang Autonomous Region tested positive for KARV, while the 57 rodent species captured in the other 6 provinces tested negative.^[21]^ The positive KARV results were solely found in great gerbils from the Xinjiang region, implying that the virus’s geographical distribution in China may be comparatively limited. This finding also emphasizes the potentially crucial role of great gerbils as key KARV hosts in Xinjiang’s specific ecological environment.

### 5.2. Gene sequencing techniques

In 2019, a study carried out in China’s Xinjiang region utilized next-generation sequencing technology to conduct an in-depth analysis of 12 spleen samples from 4 predominant wild small animal species (*Mus musculus*, *Rhombomys opimus*, *Meriones libycus*, and *Spermophilus erythrogenys*). KARV was solely detected in *Rhombomys opimus*. Employing 945 high-quality sequencing reads, the research team successfully assembled the complete genome sequence of the KARV strain DLT97.^[21]^ This study signified the first confirmation of KARV presence in China, unveiling its genomic composition and providing a pivotal reference for comprehending KARV transmission and evolution mechanisms. Phylogenetic analysis further demonstrated that KARV is closely related to Gabek Forest virus (GenBank: KF297903-KF297905) and Ntepes virus (GenBank: MT625964-MT625966) and forms a well-supported evolutionary clade with Iranian KARV (GenBank: KF297912-KF297914), implying genetic diversity within KARV. Furthermore, phylogenetic analysis based on the L gene segment demonstrated that currently known KARV sequences can be categorized into 2 major evolutionary lineages, providing a new perspective for comprehending its evolutionary characteristics and host interactions.^[21]^

## 6. Neutralizing antibody detection

Despite the unclear pathogenicity of KARV, specific antibodies have been detected in humans and other vertebrates in countries including Pakistan^[20]^ and Bangladesh.^[24]^ Apart from West Asian countries, KARV antibodies have also been detected in great gerbils and humans in African and Central Asian countries.^[25]^ The transmission cycle of KARV in these regions has been confirmed by combining the results of KARV molecular detection in various Iranian provinces with earlier molecular and serological studies.^[11,26]^ A comparison of KARV neutralizing antibody prevalence among residents of Iran’s Isfahan and Khorasan provinces between 1960 and 1975 revealed that the antibody positivity rate increased with age in both provinces. The results from Isfahan province indicated that most residents had been infected with KARV during childhood.^[7,19]^ During the summer of 1975, blood samples were collected from local residents, livestock, and great gerbils in the villages of Dormian, Shahpurabad, Komshetcheh, and Ali-Abad in Isfahan province, where sandfly fever was prevalent. In the sandfly fever epidemic areas of Iran, serological tests were performed using the plaque reduction neutralization test for 5 sandfly fever virus serotypes (Naples, Sicilian, Karimabad, Sabihaabad, and 1-47). The results demonstrated that among the 402 tested residents, the overall positivity rate for KARV antibodies was 66%. Among the 38 tested great gerbils, 32% had KARV neutralizing antibodies, suggesting once again that great gerbils may serve as hosts or amplifying hosts for KARV.^[13,19]^

## 7. Animal models

Animal models are indispensable tools for investigating disease mechanisms, progression, and evaluating prevention and control strategies. However, the predominantly subclinical nature of KARV infections has resulted in a scarcity of animal model studies. Consequently, we refer to studies of Rift Valley fever phlebovirus (RVFV), a virus in the same genus as KARV.^[27]^ In general, animal models play a crucial role in simulating human diseases and assessing pathological and immune responses, especially regarding hematological parameter changes. For RVFV specifically, rodent models are the preferred choice due to their logistical advantages and consistent genetic background. Rats and mice serve as the key models for investigating liver injury and neurological disease mechanisms.^[28]^ Moreover, the establishment of hamster models has provided new insights into the study of liver lesions caused by viral infections.^[29]^ To simulate the natural infection process, researchers typically employ intranasal injection or aerosol exposure to infect these animals.^[30–34]^ Cartwright et al utilized the footpad infection method to more realistically simulate the natural infection of humans with RVFV.^[35]^ Studies have revealed that CC057 strain mice are suitable for investigating encephalitis following footpad infection, while Sprague Dawley rats are more appropriate for studying ocular lesions.^[36]^ In the study of virus-induced abortion, pregnant rat models display pathological processes similar to those observed in pregnant women.^[37]^ Ruminant models, such as sheep, goats, and cattle, are suitable for vaccine research due to their physiological and immunological similarities to humans.^[38]^ The liver necrosis and abortion phenomena observed in animal models following infection during pregnancy aid in investigating the virus’s pathogenic mechanisms. The immune responses generated by animal models following vaccine inoculation are also crucial indicators for assessing vaccine safety and efficacy. Non-human primate models, owing to their high physiological and immunological similarities to humans, are effective models for investigating the impact of viruses on human health. African green monkeys and marmosets display significant neurological clinical symptoms in RVFV infection models and are suitable models for investigating viral diseases of the central and peripheral nervous systems.^[39–41]^ In contrast, rhesus macaques have a lower incidence of neurological diseases and are not suitable for studying the virus’s neuroinvasiveness.^[42]^ Ferrets are an emerging animal model; by intranasal inoculation of RVFV, the ferret model can simulate the natural exposure of humans to the virus through the respiratory route, investigate how the virus invades the central nervous system, and explore the mechanisms of the virus crossing the blood-brain barrier and the process of inducing neuropathological changes.^[43]^ When employing RVFV animal models for KARV research, it is essential to consider the differences between the 2 viruses in terms of genome, antigenicity, host interactions, and other factors, and to adjust or develop new animal models to ensure the accuracy and relevance of the research.

## 8. Phylogenetic relationship

**Figure 5.**
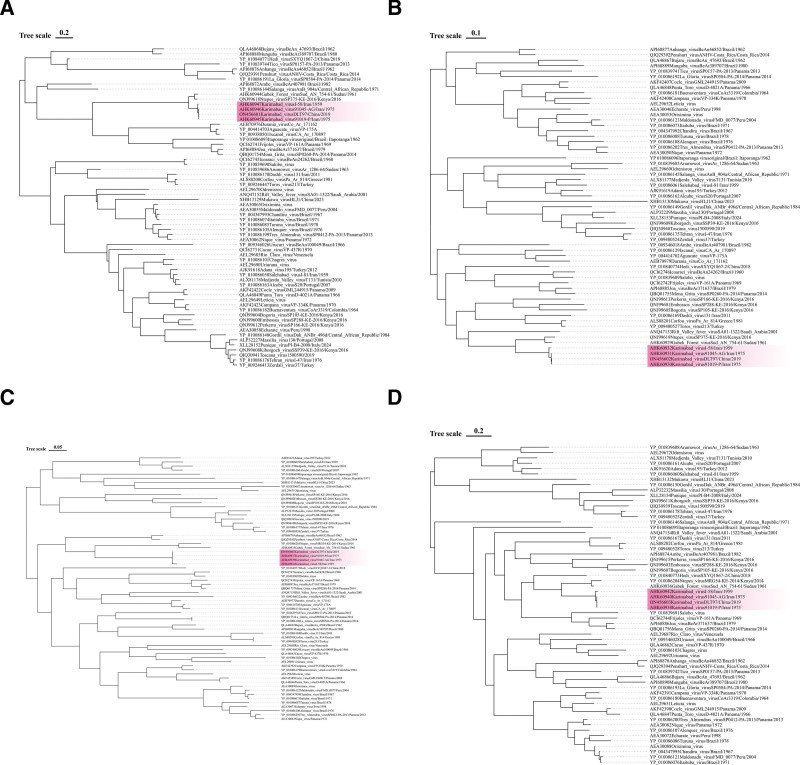
Phylogenetic analysis of Karimabad virus (KARV) and related phleboviruses based on deduced amino acid sequences. The trees were constructed using the maximum-likelihood method in MEGA5.2 with 1000 bootstrap replicates, and bootstrap values >70% are indicated at the nodes. Panel (A) shows the tree for the RNA-dependent RNA polymerase (RdRp) encoded by the L segment, (B) displays the glycoproteins (Gn and Gc) encoded by the M segment, (C) presents the nucleocapsid protein (N) encoded by the S segment, and (D) illustrates the nonstructural protein (NSs) encoded by the S segment. The current KARV strain is highlighted in red, and scale bars represent the number of amino acid substitutions per site.

To further investigate the phylogenetic relationships among KARV strains and related viruses, phylogenetic trees were constructed using the maximum-likelihood method based on the deduced amino acid sequences of all 4 viral proteins (RdRp, Gn/Gc, N, NSs) (Fig. 5). The analysis revealed that all known KARV strains, including the prototype Iranian strains (I-58/1959, 91019-P/1975, 91045-AG/1975) and the recently identified Chinese strain (DLT97/2019), form a tight, well-supported monophyletic clade across all gene segments, confirming their genetic relatedness despite geographical and temporal separation.

Pairwise comparisons of all available KARV genome sequences revealed that the KARV amino acid sequences derived from great gerbils in China between 2019 and 2020 clustered closely with the KARV sequences detected in *Phlebotomus papatasi* from Iran in 1975 and *Phlebotomus* spp. in 1959.This cohesive KARV clade was most closely related to, yet distinctly separate from, a clade containing Gabek Forest virus (isolated in Sudan) across all phylogenetic analyses (Fig. 5).

Concurrently, the KARV strains isolated in Iran in 1975 demonstrated significant genetic divergence compared to the strains isolated in Iran in 1959, with nucleotide sequence identity of 89.4%, 90.7%, and 88.0% for the L, M, and S segments, respectively. The amino acid sequence similarity of 99.4%, 99.1%, 99.2%, and 98.9% for the L, M, N, and NSs proteins, respectively. In contrast, the Chinese KARV strains, when compared to the strains isolated in Iran in 1959, exhibited nucleotide sequence identity of 93.9%, 80.1%, and 81.6% for the L, M, and S segments, respectively. The amino acid sequence similarity of 99.2%, 88.9%, 99.1%, and 92.8% for the L, M, N, and NSs proteins, respectively (Fig. 2). Moreover, sequence analysis of KARV strains isolated from great gerbils in the Xinjiang region of China confirmed their placement within the KARV cluster. The sequence identity to Iranian KARV ranged from 79.1 to 93.9%, with some variations in the L, M, and S segments. These 2 viruses exhibited cross-reactivity with most Naples sandfly fever complex viruses in hemagglutination inhibition tests but did not cross-react in complement fixation tests, indicating their close relationship and distinguishability.^[8]^ The biological characteristics of KARV hosts and the involvement of their immune systems may contribute to viral gene mutations or rearrangements, resulting in substantial genetic variations among KARV strains.

## 9. Future perspectives

Although significant progress has been made in KARV research in recent years, many questions remain to be further explored and answered. Central to these uncertainties is KARV’s capacity to infect phylogenetically distant hosts – from rodents to humans – while maintaining low apparent pathogenicity, a paradox that may stem from its glycoprotein adaptability and utilization of evolutionarily conserved entry pathways.

Firstly, the pathogenic mechanisms underlying KARV infection remain elusive. While studies have demonstrated that KARV possesses the ability to infect humans and cause symptomatic diseases, its pathogenic process, clinical manifestations, and potential complications necessitate further investigation. The establishing of suitable animal models and the execution of pathological and immunological studies will facilitate the elucidation of the molecular mechanisms underlying KARV infection and host responses.

Secondly, the transmission dynamics and epidemiological features of KARV require further clarification. As the primary transmission vector of KARV, the biological characteristics, seasonal distribution, and habitat preferences of sandflies, as well as the influence of these factors on virus transmission, warrant further investigation. Furthermore, rodents, as natural hosts of KARV, play a crucial role in maintaining the natural cycle of the virus; however, different rodent populations may exhibit varying different susceptibilities and transmission capabilities for KARV, which necessitates confirmation through field investigations and experimental studies.

Thirdly, the genetic diversity and evolutionary mechanisms of KARV are noteworthy. Studies have revealed that KARV possesses distinct evolutionary branches, however the extent of its genetic variation, spatiotemporal dynamics, and relationship with host interactions remain unclear. The execution of large-scale viral genome sequencing and phylogenetic analysis will facilitate the revelation of the evolutionary history and adaptation mechanisms of KARV and enable the prediction of its future evolutionary trajectories.

Lastly, the development of diagnostic methods and control strategies for KARV constitutes a focal point of future research endeavors. Although serological and molecular biological methods are currently available for KARV detection, there remains a pressing need to develop more rapid, sensitive, and specific diagnostic technologies to fulfill the requirements for early identification and timely response. Future research should embrace a multidisciplinary approach, fortify the integration of basic and applied research, enhance the level of knowledge and control capabilities for KARV, and proactively prepare for the potential public health challenges it may pose. A major limitation in understanding KARV evolution is the scarcity of genomic data. Currently, only 3 complete genomes are available globally (two from Iran, one from China). This paucity severely limits phylogeographic resolution. Urgent priority must be given to sequencing archived samples from seropositive regions like Sudan and Pakistan to bridge the gap between serological evidence and genomic characterization.

## Author contributions

**Conceptualization:** Kai Guo.

**Data curation:** Yingxin Tu.

**Investigation:** Anan Wang.

**Methodology:** Kai Guo, Anan Wang.

**Project administration:** Jian Song.

**Software:** Meixi Ren, Tao Xu.

**Validation:** Meixi Ren, Tao Xu.

**Visualization:** Meixi Ren.

**Writing – original draft:** Yingxin Tu.

**Writing – review & editing:** Guoyu Niu.
